# Supplementary material for: Potential Correlation between Changes in Serum FGF21 Levels and Lenvatinib-Induced Appetite Loss in Patients with Unresectable Hepatocellular Carcinoma
Source: Cancers (Basel). 2023 Jun 20;15(12):3257. doi: 10.3390/cancers15123257 (PMC10296590; doi:10.3390/cancers15123257)
Supplement: Supplementary file 1 [file cancers-15-03257-s001.zip › supplementary_Figure_S1.pptx]

## Slide 1
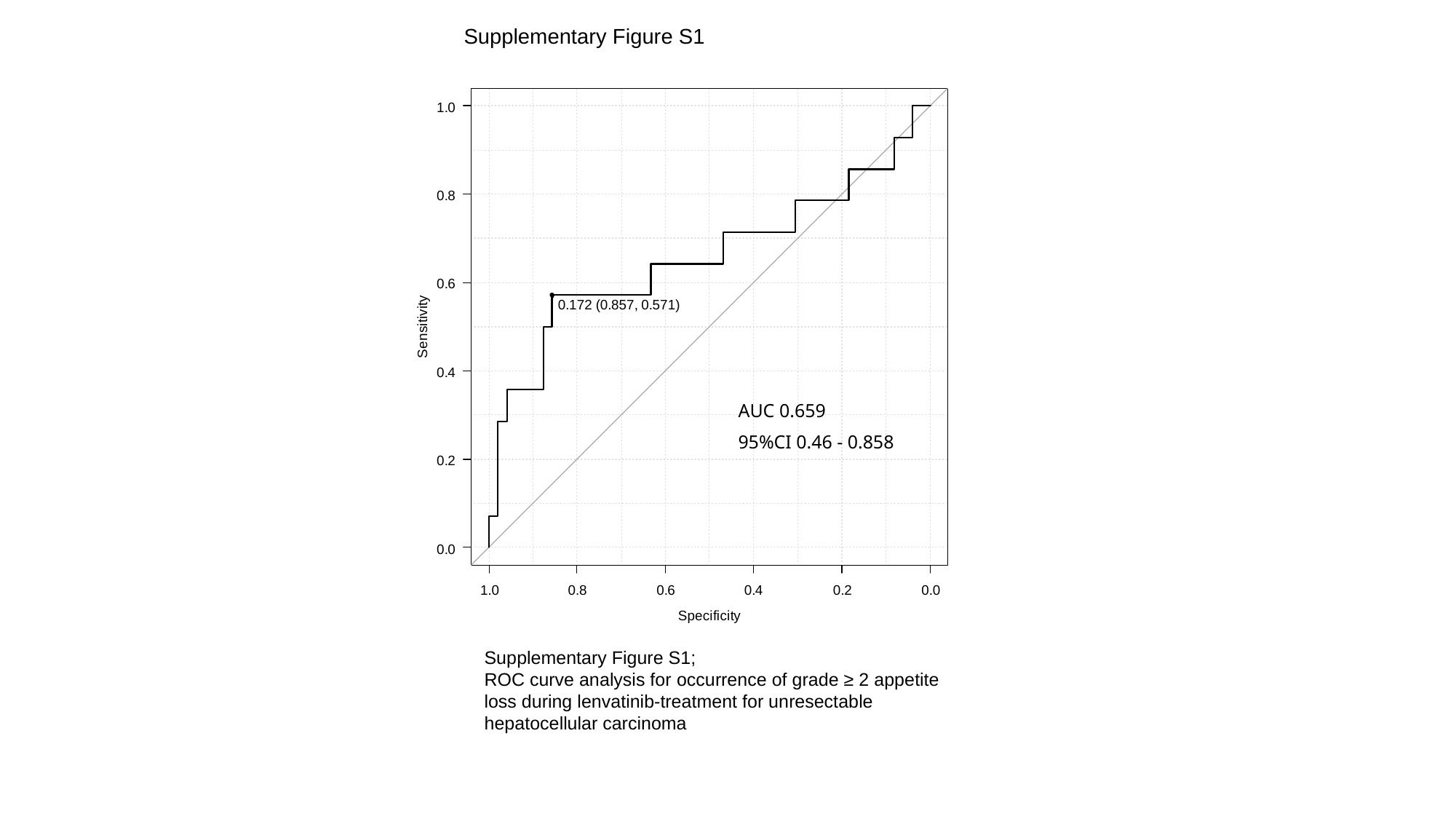

Supplementary Figure S1
AUC 0.659
95%CI 0.46 - 0.858
Supplementary Figure S1;
ROC curve analysis for occurrence of grade ≥ 2 appetite loss during lenvatinib-treatment for unresectable hepatocellular carcinoma
