# Supplementary material for: Potential Correlation between Changes in Serum FGF21 Levels and Lenvatinib-Induced Appetite Loss in Patients with Unresectable Hepatocellular Carcinoma
Source: Cancers (Basel). 2023 Jun 20;15(12):3257. doi: 10.3390/cancers15123257 (PMC10296590; doi:10.3390/cancers15123257)
Supplement: Supplementary file 1 [file cancers-15-03257-s001.zip › Supplementary_Table_S1.pdf]

Supplementary Table S1

Multivariate logistic regression analysis of factors associated with grade 2 or more appetites loss during lenvatinib for unresectable hepatocellular carcinoma

	Univariate analysis (p value)	Multivariate analysis (p value)	Odds ratio
**Delta FGF21**	**0.009**	**0.037***	**1.010 (1.001–1.020)**
TNM classification	0.144	0.889	1.113 (0.987–1.255)
Body mass index	0.136	0.082	1.067 (0.429–2.655)

Abbreviations: Delta FGF21, rate of changes in FGF21 between baseline and at 4 weeks after Lenvatinib; HCC, hepatocellular carcinoma; *Statistically significant difference, $P < 0.05$
